# Aging‐associated changes in CD47 arrangement and interaction with thrombospondin‐1 on red blood cells visualized by super‐resolution imaging

**DOI:** 10.1111/acel.13224

**Published:** 2020-08-31

**Authors:** Feng Wang, Yan‐Hou Liu, Ting Zhang, Jing Gao, Yangyue Xu, Guang‐Yao Xie, Wen‐Jie Zhao, Hongda Wang, Yong‐Guang Yang

**Affiliations:** ^1^ Key Laboratory of Organ Regeneration and Transplantation of the Ministry of Education The First Hospital Institute of Immunology Jilin University Changchun China; ^2^ National‐local Joint Engineering Laboratory of Animal Models for Human Diseases Changchun China; ^3^ State Key Laboratory of Electroanalytical Chemistry Changchun Institute of Applied Chemistry Chinese Academy of Sciences Changchun China; ^4^ International Center of Future Science Jilin University Changchun China

**Keywords:** aging, CD47, dSTORM, red blood cells, thrombospondin‐1

## Abstract

CD47 serves as a ligand for signaling regulatory protein α (SIRPα) and as a receptor for thrombospondin‐1 (TSP‐1). Although CD47, TSP‐1, and SIRPα are thought to be involved in the clearance of aged red blood cells (RBCs), aging‐associated changes in the expression and interaction of these molecules on RBCs have been elusive. Using direct stochastic optical reconstruction microscopy (dSTORM)‐based imaging and quantitative analysis, we can report that CD47 molecules on young RBCs reside as nanoclusters with little binding to TSP‐1, suggesting a minimal role for TSP‐1/CD47 signaling in normal RBCs. On aged RBCs, CD47 molecules decreased in number but formed bigger and denser clusters, with increased ability to bind TSP‐1. Exposure of aged RBCs to TSP‐1 resulted in a further increase in the size of CD47 clusters via a lipid raft‐dependent mechanism. Furthermore, CD47 cluster formation was dramatically inhibited on *thbs1* ^−/−^ mouse RBCs and associated with a significantly prolonged RBC lifespan. These results indicate that the strength of CD47 binding to its ligand TSP‐1 is predominantly determined by the distribution pattern and not the amount of CD47 molecules on RBCs, and offer direct evidence for the role of TSP‐1 in phagocytosis of aged RBCs. This study provides clear nanoscale pictures of aging‐associated changes in CD47 distribution and TSP‐1/CD47 interaction on the cell surface, and insights into the molecular basis for how these molecules coordinate to remove aged RBCs.

## Graphical abstract

On young RBCs (Top), CD47 molecules reside as nanoclusters with minimal binding to TSP‐1 trimmers. On aged RBCs (Bottom), CD47 proteins form bigger and denser clusters and gain increased ability to bind TSP‐1, thus promoting phagocytosis of aged RBCs.

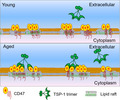

## 1 INTRODUCTION

The removal of apoptotic or senescent cells is necessary for physiological development and homeostasis. Generally, the equilibrium between “eat me” and “don't eat me” determines whether a cell will be cleared by phagocytes (Oldenborg, 2013). CD47 is expressed on the surface of most cell types and provides an anti‐phagocytic ("don't eat me") signal by binding to signal regulatory protein α (SIRPα), an inhibitory receptor on macrophages (Oldenborg, 2013; Oldenborg, Gresham, & Lindberg, 2001). Apoptotic cells have a reduced level (Gardai et al., 2005; Khandelwal, van Rooijen, & Saxena, 2007) or altered pattern (Burger, Hilarius‐Stokman, de Korte, van den Berg, & van Bruggen, 2012; Gardai et al., 2005; Lv et al., 2015) of CD47 expression, rendering the CD47/SIRPα inhibitory signal ineffective. Studies using cancer cells suggested that alterations in the spatial distribution of CD47 during apoptosis may contribute to apoptotic cell clearance (Gardai et al., 2005). It has been reported that CD47 molecules on Jurkat T cells are evenly distributed under normal conditions but become grouped into patches during apoptosis, leaving apoptotic signals such as calreticulin and phosphatidylserine exposed to trigger phagocytosis(Gardai et al., 2005). In contrast, CD47 distribution on human colorectal adenocarcinoma cells was reported to change from clustered to a diffuse pattern during apoptosis, which is associated with loss of binding to SIRPα, leading to increased phagocytosis (Lv et al., 2015). One possible explanation for these disparate observations in cancer cells is that the diffraction‐limited resolution of conventional fluorescence microscopy is insufficient to provide structural insights into nanoscale protein clusters.

The removal of aged or senescent red blood cells (RBCs) is necessary for blood turnover and alterations, either exacerbation or suppression, in this process can cause serious health problems (Patel, Patel, & Higgins, 2015; Pernow, Mahdi, Yang, & Zhou, 2019). For decades, Band 3 (Lutz, 2012), oxidative stress, phosphatidylserine (Freikman & Fibach, 2011), and CD47 have gained great attentions for their potential role in RBC lifespan determination (Arias, 2017; Burger et al., 2012; Khandelwal et al., 2007). Thrombospondin‐1 (TSP‐1), a soluble ligand for CD47, regulates cell motility, proliferation, differentiation, and apoptosis (Adams & Lawler, 2011). A previous study suggests that CD47 on experimentally aged RBCs undergoes a conformational change upon binding to TSP‐1‐ or TSP‐1‐derived peptide 4N1K, and that this altered CD47 expression and binding to TSP‐1 provide a pro‐phagocytic signal (Burger et al., 2012). Coincidently, a conformational change in CD47, as identified by anti‐CD47 mAb 2D3 on sickle RBCs (Brittain, Mlinar, Anderson, Orringer, & Parise, 2001), is also associated with a gain in TSP‐1 binding capacity (Brittain et al., 2001). These studies suggest that the expression pattern of CD47 on RBCs might be important in determining its TSP‐1 binding capacity and that TSP‐1/CD47 interaction may promote phagocytosis by affecting CD47‐SIRPα signaling or providing pro‐phagocytic signals. However, the precise changes on aged RBCs in CD47 expression and conformation that confer TSP‐1 binding remain to be determined.

By breaking the diffraction limit, single‐molecule localization microscopy provides a new tool for analyzing the spatial organization of molecules at the nanoscale level. Beyond producing attractive images with nanometer resolution, super‐resolution imaging is evolving toward quantitative single‐molecule localization microscopy (qSMLM) (Annibale, Vanni, Scarselli, Rothlisberger, & Radenovic, 2011; Durisic, Laparra‐Cuervo, Sandoval‐Alvarez, Borbely, & Lakadamyali, 2014; Endesfelder & Heilemann, 2014). Using direct stochastic optical reconstruction microscopy (dSTORM) and related quantitative analysis, we characterized aging‐associated changes in the number and expression pattern of CD47 on murine RBCs at the nanoscale level, and the correlation of these changes in CD47 with its TSP‐1 binding capacity. We also examined the potential role of TSP‐1/CD47 binding in inducing CD47 protein rearrangement and removal of aged RBCs, and the involvement of lipid rafts in the process.

## 2 RESULTS

### 2.1 RBCs from aged mice show a decrease in the total number of CD47 molecules but an increase in the density and number of CD47 proteins per CD47 cluster

**Figure 1:**
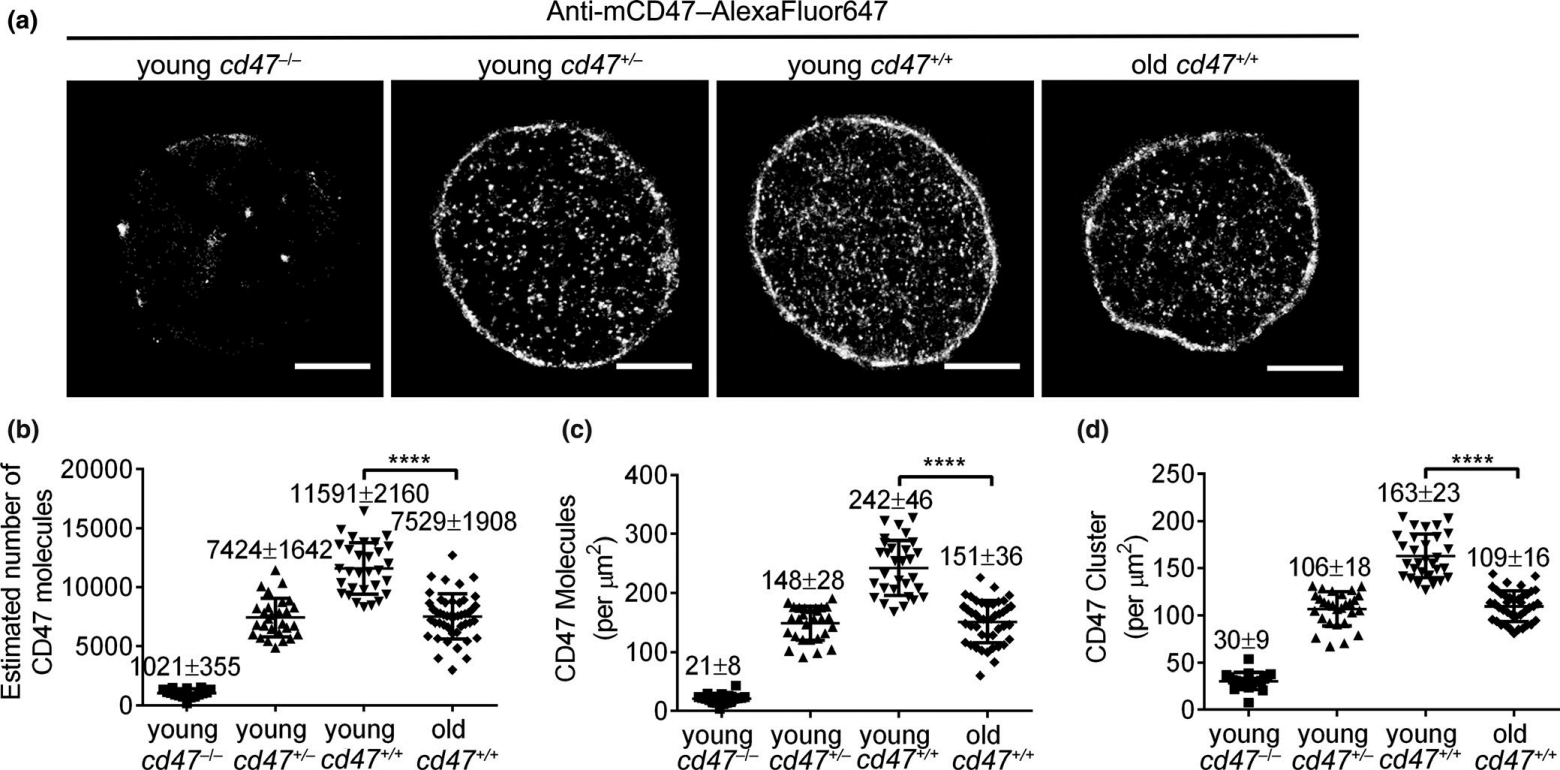
Direct STORM imaging and quantitative analysis of CD47 on young vs. old mouse RBCs. (a) Representative dSTORM images of CD47 distribution on *cd47* ^−/−^, *cd47* ^+/−^, and *cd47* ^+/+^ mouse RBCs. Scale bar = 2 μm. (b–d) Quantitative analysis of CD47 on young *cd47* ^−/−^ mouse RBCs, young *cd47* ^+/−^ mouse RBCs, young *cd47* ^+/+^ mouse RBCs, and old *cd47* ^+/+^ mouse RBCs. Data shown are estimated total numbers (b); protein densities (molecules/µm^2^) (c); and cluster densities (cluster/µm^2^) (d) of cell surface CD47. Data are combined from 3 independent experiments and presented as mean ± *SD* (young *cd47* ^−/−^ mouse RBCs, *n* = 21; young *cd47* ^+/−^ mouse RBCs, *n* = 28; young *cd47* ^+/+^ mouse RBCs, *n* = 30; old *cd47* ^+/+^ mouse RBCs, *n* = 41). ****p < 0.0001 (by two‐tailed unpaired Student's *t*‐test)

RBC senescence is a time‐dependent, irreversible, non‐linear physiological process (Kay, 2005; Lang & Qadri, 2012). Most previously used RBC aging models involve in vitro exposure of RBCs to harsh oxidative conditions that do not reflect the in vivo aging process (Burger et al., 2012; Franco, 2012). It has been shown that RBCs from old individuals age more rapidly, with a decreased half‐life and reduced hemoglobin concentrations, than those from young individuals (Glass, Gershon, & Gershon, 1983; Kay, 2005; Kosower, 1993; Kubota et al., 1991; Magnani et al., 1988), indicating that comparison of RBCs between young and old individuals may provide insights into aging‐associated changes in RBCs. Thus, to determine aging‐associated changes in CD47, we compared CD47 expression in RBCs from young (2 months of age) and old (>22 months of age) mice by dSTORM imaging, with young *cd47* ^−/−^ and *cd47* ^+/−^ mouse RBCs as controls. Compared to young *cd47* ^+/+^ mouse RBCs, old *cd47* ^+/+^ mouse RBCs had a significant decrease in the total molecule number (Figure 1a,b), protein density (Figure 1a,c), and cluster density (Figure 1a,d) of cell surface CD47, and the values of these parameters became similar to those of *cd47* ^+/−^ mouse RBCs.

**Figure 2:**
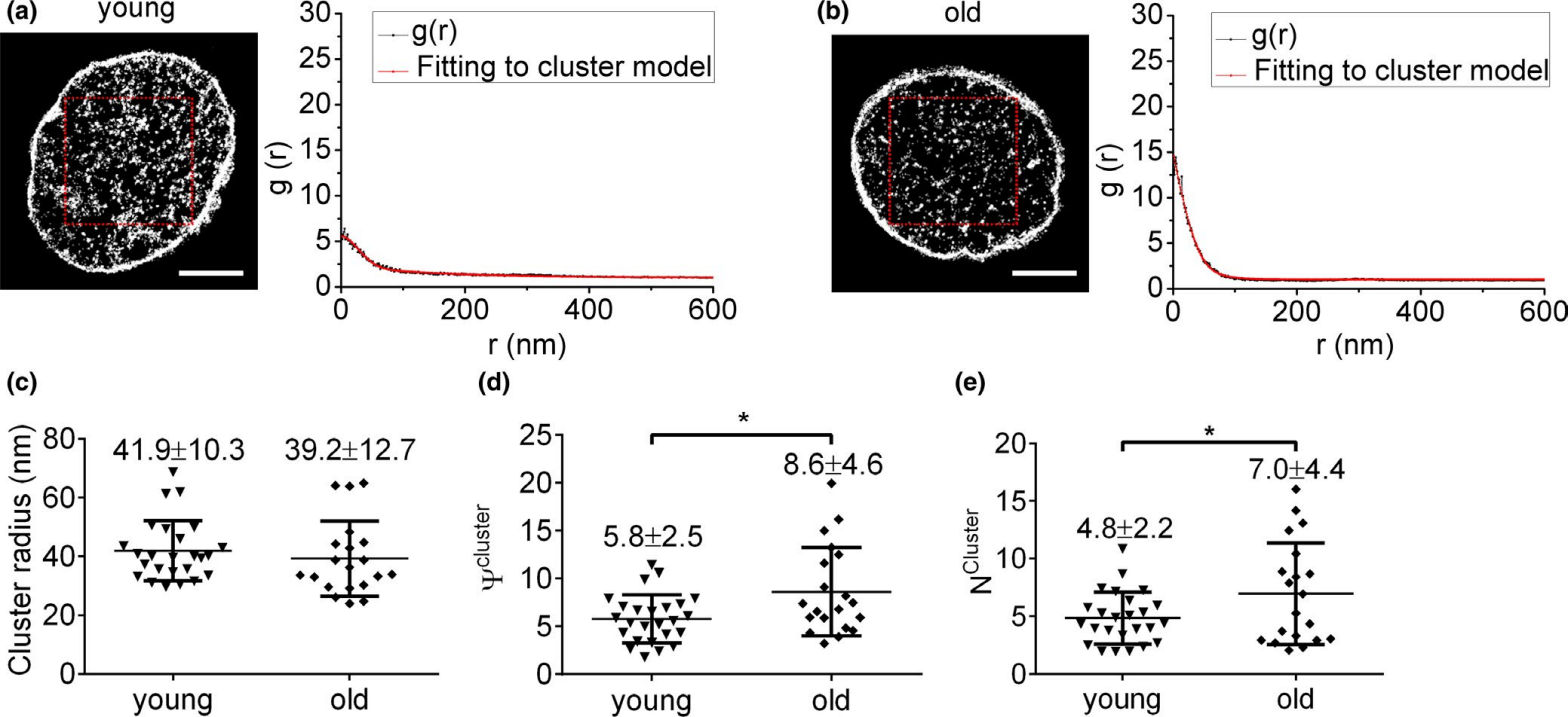
Direct STORM imaging and univariate pair correlation function analysis of CD47 on RBCs from young and old *cd47* ^+/+^ mice. (a, b) Left and right panels show RBC dSTORM images (scale bar = 2 μm) and corresponding pair correlation function analysis, respectively, for young (a) and old (b) mouse RBCs. (c–e) Clustering parameters of CD47 clusters on *cd47* ^+/+^ RBCs from young or old mice: (c) CD47 cluster radius (nm); (d) density of CD47 proteins in cluster (ψ^cluster^); (e) average numbers of CD47 proteins per cluster (N^cluster^). Unpaired *t*‐test was used; n.s., not significant; *p < 0.05. Data combined from 3 independent experiments are presented as mean ± *SD* (each symbol represents an individual cell; 20‐25 cells per group were reanalyzed)

Univariate pair correlation function analysis was performed to further characterize CD47 clusters. The pair correlation function is a well‐established cluster algorithm (Sengupta, Jovanovic‐Talisman, & Lippincott‐Schwartz, 2013; Sengupta et al., 2011; Veatch et al., 2012) that can be used to avoid repeatedly localizing the same molecule, making it preferable for dSTORM imaging data analysis(Notelaers et al., 2014; Sauer & Heilemann, 2017). Briefly, an area of 4 × 4 μm^2^ was selected (red box in reconstructed dSTORM images, left panels in Figure 2a,b) and analyzed by the PC‐PALM plug‐in toolset, and the corresponding g(r) curves were drawn according to data in the correlation field (right panels in Figure 2a,b). Three cluster parameters (CD47 cluster radius, density of CD47 proteins within a cluster (*ψ* ^cluster^), and average number of CD47 proteins per cluster (*N* ^cluster^) were obtained from the PC‐PALM fitting table (as detailed in the Materials and Methods). Although the cluster radiuses were comparable between young mouse and old mouse RBCs (Figure 2c), the latter showed a significant increase in both *ψ* ^cluster^ (*p* = 0.012; Figure 2d) and *N* ^cluster^ (*p* = 0.043; Figure 2e). These results indicate that although RBC aging is associated with a decrease in the total number of CD47 molecules, the density and average number of CD47 proteins in each CD47 cluster on old mouse RBCs were significantly increased.

### 2.2 CD47 on RBCs from aged mice shows increased binding to 4N1K

**Figure 3:**
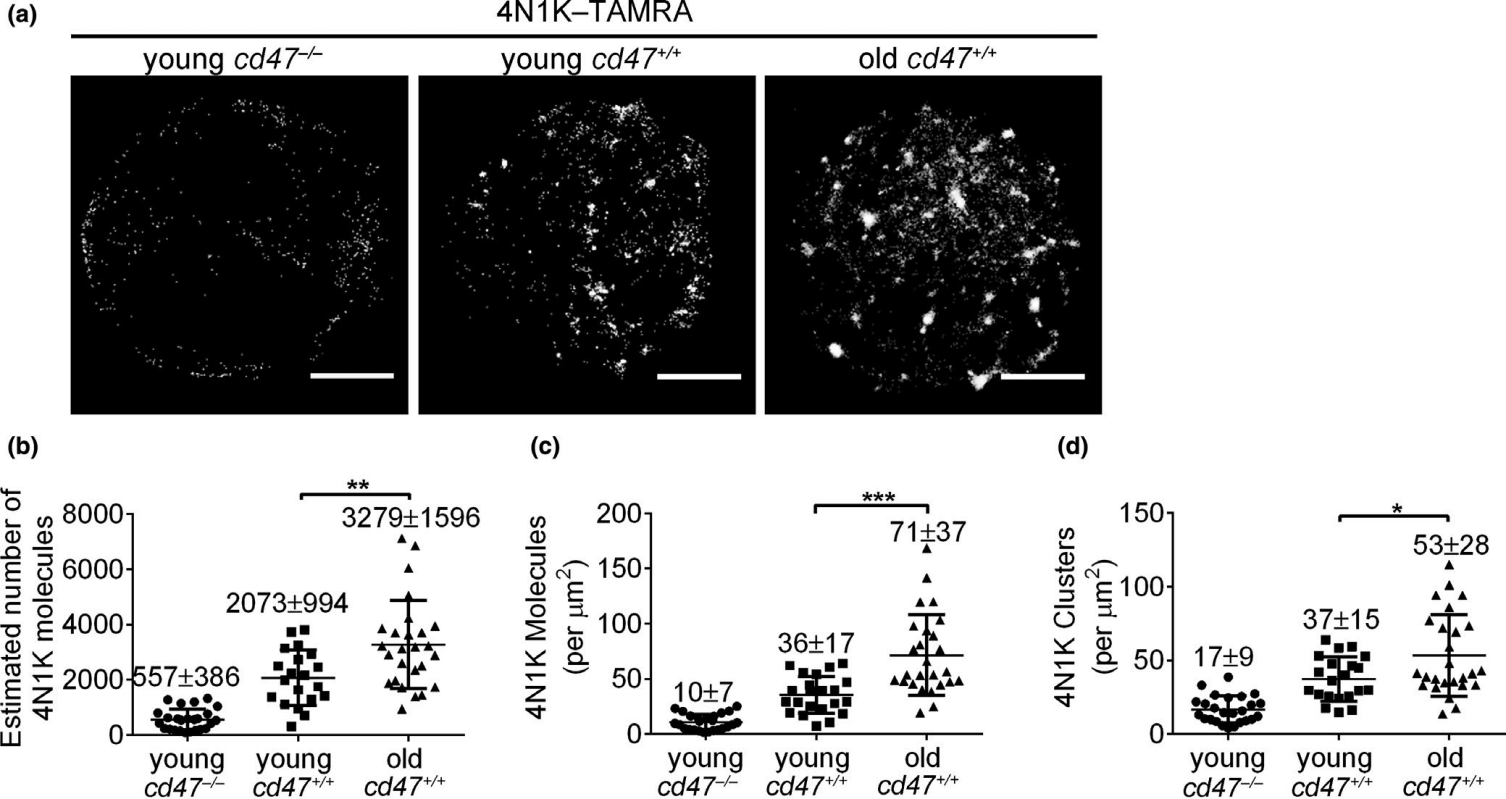
Direct STORM imaging and quantitative analysis of 4N1K binding on young vs. old mouse RBCs. (a) Representative dSTORM images of 4N1K binding on young *cd47* ^−/−^, young *cd47* ^+/+^ and old *cd47* ^+/+^ mouse RBCs; scale bar = 2 μm. (b–d) Quantitative analysis of 4N1K binding on young *cd47* ^−/−^ mouse RBCs, young *cd47* ^+/+^ mouse RBCs, and old *cd47* ^+/+^ mouse RBCs. Data shown are estimated numbers (b), protein densities (molecules/µm^2^) (c) and cluster densities (cluster/µm^2^) (d) of 4N1K molecules on RBCs. Data are combined from three independent experiments and presented as mean ± *SD* (young *cd47* ^−/−^ mouse RBCs, *n* = 25; young *cd47* ^+/+^ mouse RBCs, *n* = 21; old *cd47* ^+/+^ mouse RBCs, *n* = 26). *p < 0.05; **p < 0.01; ***p < 0.001 (by two‐tailed unpaired Student's *t*‐test)

4N1K is a TSP‐1 C‐terminal‐derived peptide (K‐_1016_RFYVVMWK_1024_‐K) that contains a Val–Val–Met motif required for TSP‐1 binding to CD47 (Gao et al., 1996), and has been widely used as a TSP‐1 functional mimic peptide (Burger et al., 2012; Tulasne et al., 2001). Because 4N1K may induce CD47‐independent non‐specific molecular aggregates when used at micromolar concentrations (Barazi et al., 2002; Leclair & Lim, 2014; Tulasne et al., 2001), 4N1K was used at a concentration of 100 nM in the experiments comparing binding of old vs. young mouse RBCs. Old *cd47* ^+/+^ mouse RBCs, despite the reduced number of CD47 molecules (Figure 1), showed significantly enhanced binding to 4N1K compared to young *cd47* ^+/+^ mouse RBCs (Figure 3a–d), while both showed comparably minimal binding to the negative control peptide 4NGG (Figure S1). The estimated molecule number (Figure 3b), molecular density (Figure 3c), and cluster density (Figure 3d) of 4N1K on RBCs from old *cd47* ^+/+^ mice increased by 60%, 97%, and 43%, respectively, compared to those from young *cd47* ^+/+^ mice. We also compared 4N1K binding to young vs. aged RBCs from young mice using an *in vivo* biotinylation method (Khandelwal et al., 2007; Saxena, Bhardwaj, Sachar, Puri, & Khandelwal, 2012). Young RBCs (biotin^−^) and aged RBCs (biotin^+^) were sorted from young mice (2 months of age) at day 20 post‐biotinylation. dSTORM imaging revealed markedly increased CD47 clustering in aged RBCs compared to young RBCs (Figure S2a‐b). Furthermore, aged RBCs showed significantly greater binding to 4N1K than young RBCs (Figure S2c‐e). Together, these results indicate that the binding of 4N1K on RBCs significantly increased during the aging process.

### 2.3 4N1K induces hierarchical rearrangement of CD47 on aged RBCs

**Figure 4:**
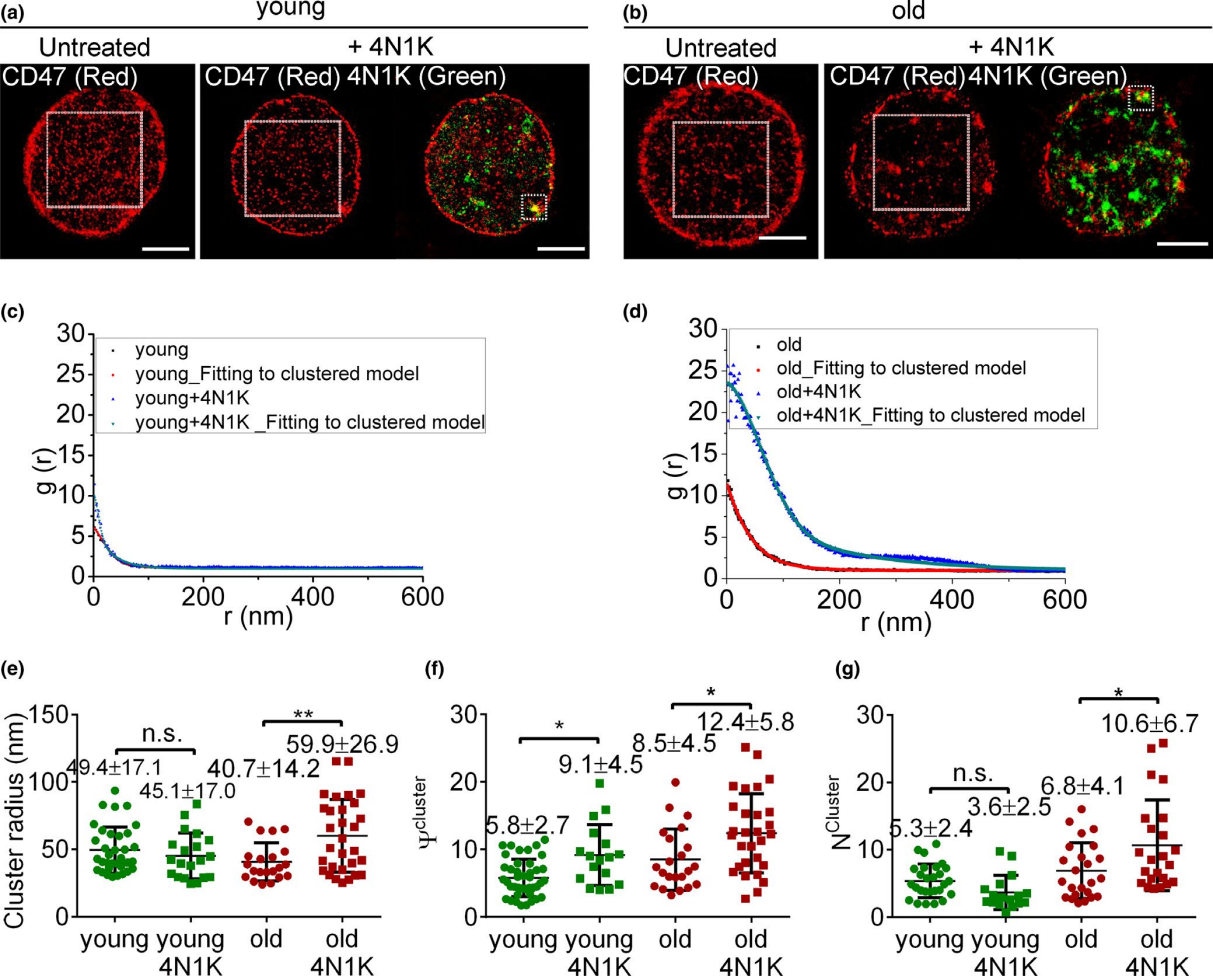
Two‐color dSTORM imaging and cluster characteristics of CD47 on young vs. old mouse RBCs after 4N1K treatment. (a, b) Representative dSTORM images of CD47 distribution (red) on untreated or 4N1K‐treated young (a) and old (b) *cd47* ^+/+^ mouse RBCs. Scale bar = 2 μm. For both untreated and 4N1K‐treated samples, an area of 4 × 4 μm^2^ was selected, and CD47 distribution characteristics were analyzed with pair correlation function (corresponding g(r) curves of young and old mouse RBCs are shown in c and d, respectively). (c, d) Univariate pair correlation function analysis of CD47 distribution on untreated and 4N1K‐treated young (c) or old (d) *cd47* ^+/+^ mouse RBCs. (e–g) Shown are cluster radius (e), ψ^cluster^ (f) and N^cluster^ (g) of CD47 clusters on *cd47* ^+/+^ RBCs from young or old mice that were untreated or treated with 4N1K. Data from 3 independent experiments are combined and presented as mean ± *SD* (untreated young mouse RBCs, *n* = 41; young mouse RBCs treated with 4N1K, *n* = 17; untreated old mouse RBCs, *n* = 24; old mouse RBCs treated with 4N1K, *n* = 23). n.s. indicates not significant; *p < 0.05; **p < 0.01 (by two‐way ANOVA with Tukey's correction)

To further confirm that CD47 is the binding target of 4N1K on RBCs, we conducted dual‐color dSTORM imaging to simultaneously visualize 4N1K and CD47. RBCs were first incubated with 4N1K–TAMRA (green) or PBS, and then stained with anti‐CD47–AF647 (red). For young mouse RBCs, CD47 molecules were sparsely bound to 4N1K and remained evenly distributed in small clusters after 4N1K treatment (Figure 4a). In contrast, CD47 molecules (red) on the old mouse RBCs were spatially aggregated in large clusters with abundant 4N1K (green) binding (Figure 4b). Furthermore, 4N1K treatment induced a significant increase in cluster radius (Figure 4e), *ψ* ^cluster^ (Figure 4f), and *N* ^cluster^ in old mouse RBCs (Figure 4g), while only a moderate increase in *ψ* ^cluster^ was detected in young mouse RBCs following 4N1K treatment (Figure 4f). These data indicate that CD47 molecules on old mouse RBCs bind to TSP‐1 peptides with greater affinity, which in turn causes CD47 molecules to rearrange into larger clusters.

### 2.4 CD47 on RBCs from aged mice shows increased binding to TSP‐1

**Figure 5:**
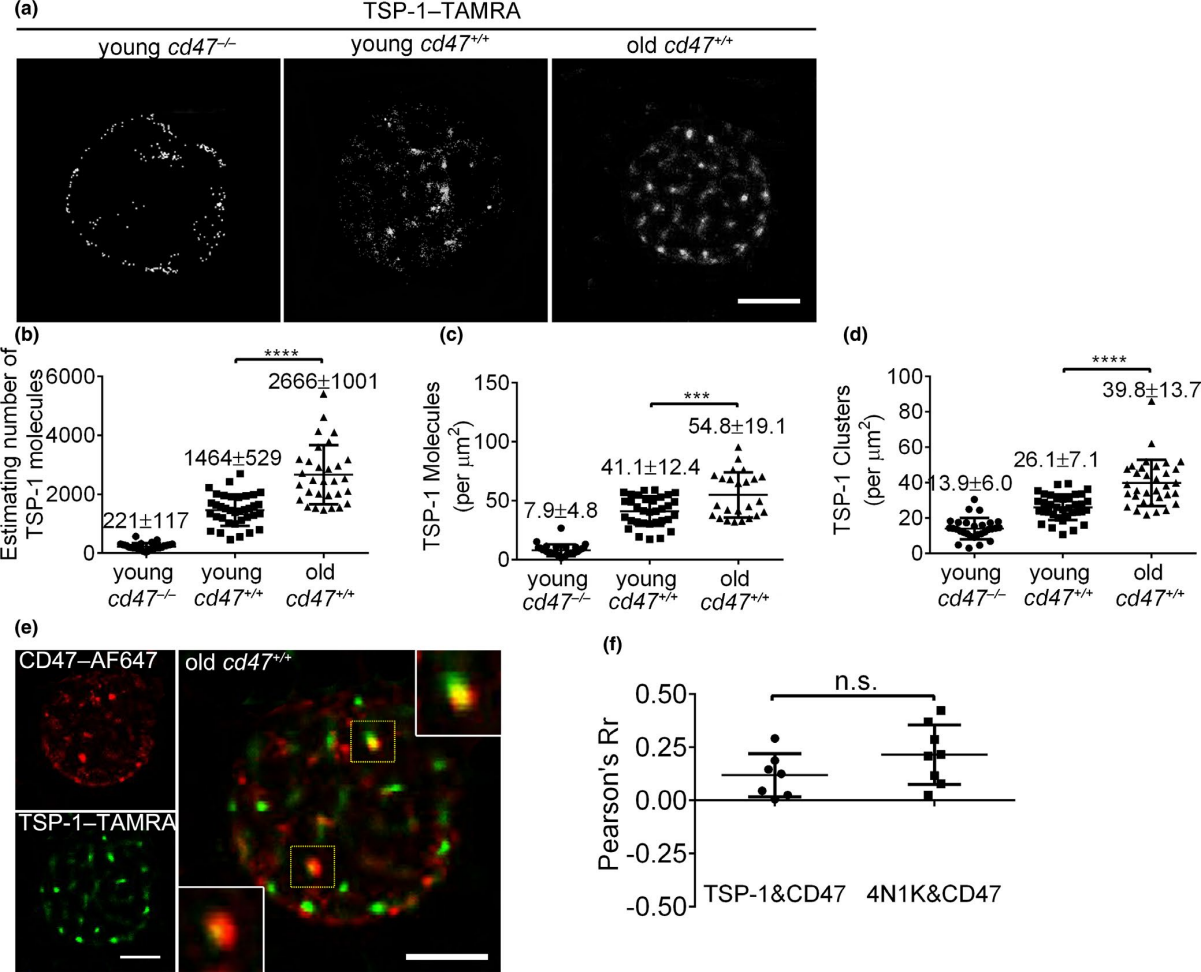
Direct STORM imaging and quantitative analysis of TSP‐1 binding on young vs. old mouse RBCs. (a) Representative dSTORM images of TSP‐1 binding on the indicated mouse RBCs. Scale bar = 2 μm. (b–d) Quantitative analysis of TSP‐1 binding on young *cd47* ^−/−^, young *cd47* ^+/+^, and old *cd47* ^+/+^ mouse RBCs. Data shown are estimated numbers (b), protein densities (molecules/μm^2^) (c), and cluster densities (cluster/μm^2^) (d) of TSP‐1 molecules. Data are combined from three independent experiments and presented as mean ± *SD* (young *cd47* ^−/−^ mouse RBCs, *n* = 30; young *cd47* ^+/+^ mouse RBCs, *n* = 36; old *cd47* ^+/+^ mouse RBCs, n = 30). ***p < 0.001; ****p < 0.0001 (by two‐tailed unpaired Student's *t*‐test). (e) Two‐color dSTORM images showing co‐localization of TSP‐1–TAMRA with CD47–AF647 on *cd47* ^+/+^ old RBCs. High magnification images (insets) of co‐localized TSP‐1 and CD47 clusters are shown (scale bar = 2 μm). (f) Pearson coefficient (Pearson's Rr) showing levels of co‐localization between CD47 with TSP‐1 (0.12 ± 0.09; *n* = 6) or with 4N1K (0.22 ± 0.14; *n* = 8) on old *cd47* ^+/+^ RBCs

The observations made with 4N1K were further validated by comparing the binding of fluorescence‐conjugated TSP‐1 (TSP‐1–TAMRA) on young vs. old *cd47* ^+/+^ mouse RBCs, with young *cd47* ^−/−^ RBCs used as controls. Like 4N1K peptides, TSP‐1 showed minimal (background) binding to *cd47* ^−/−^ RBCs, whereas TSP‐1 binding to *cd47* ^+/+^ RBCs was clearly detected with a significantly greater level on old versus young mouse RBCs (Figure 5a–d). When compared to young *cd47* ^+/+^ mouse RBCs, the estimated molecule number, molecule density, and cluster density of TSP‐1 bound to old mouse RBCs increased by approximately 80% (Figure 5b), 34% (Figure 5c), and 54% (Figure 5d), respectively. Dual‐color dSTORM imaging revealed that TSP‐1 was directly bound to CD47 on old mouse RBCs (Figure 5e). Furthermore, by using the co‐localization finder plug‐in for ImageJ, we confirmed that the levels of TSP‐1/CD47 co‐localization on old mouse RBCs were similar to that of 4N1K with CD47 (Figure 5f). These results are consistent with the experiments using 4N1K, confirming that CD47 proteins on old mouse RBCs have an increased ability to bind TSP‐1.

### 2.5 TSP‐1 is essential in aging‐associated CD47 aggregation on RBCs and is involved in the clearance of aged RBCs

**Figure 6:**
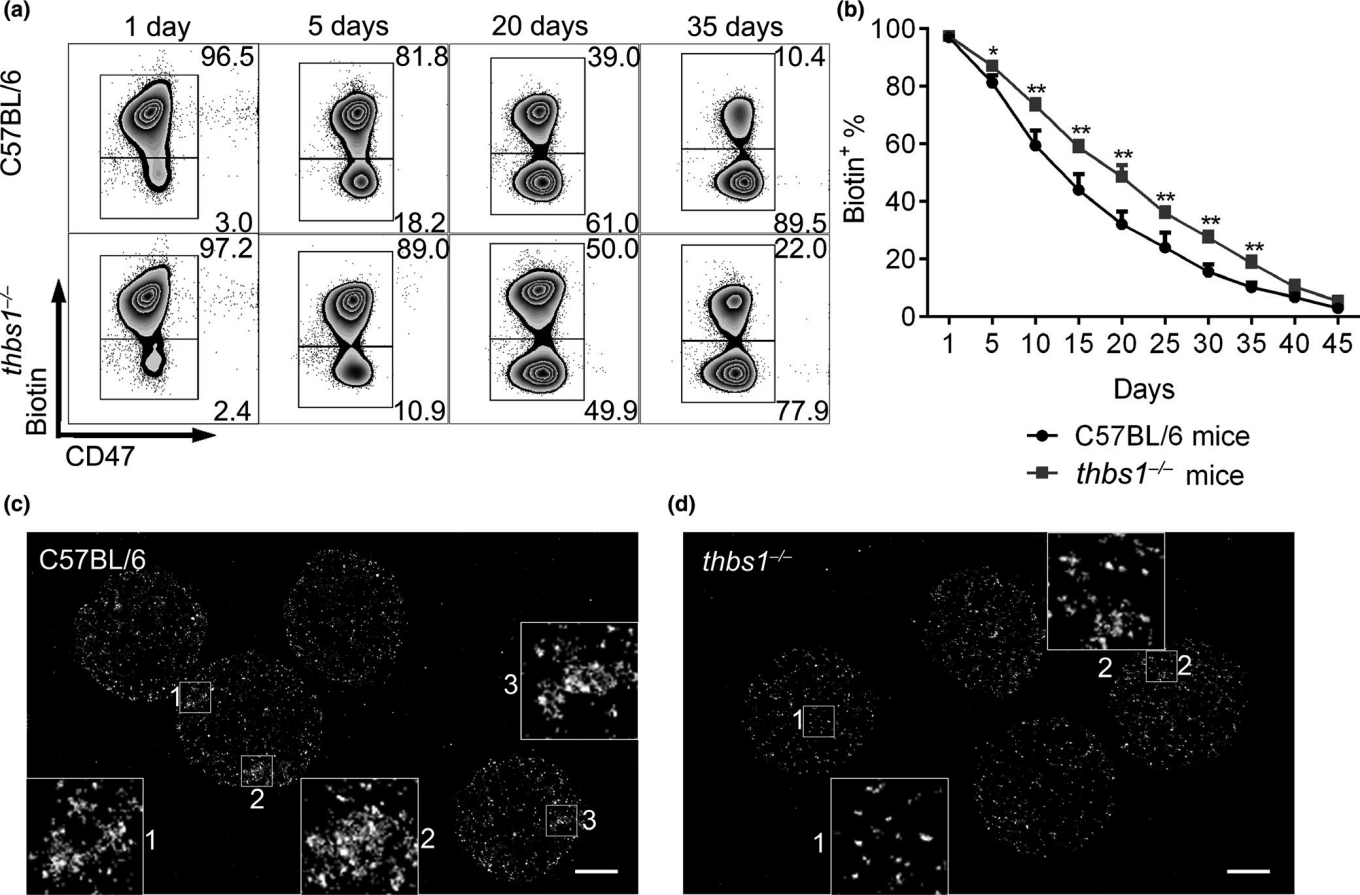
RBC life span and CD47 distribution pattern in old WT and *thbs1* ^−/−^ mice. Circulating RBCs in 18 months of age WT or *thbs1* ^−/−^ mice were labeled by biotinylation as described under Materials and Methods. Blood cells were collected at 1, 5, 10, 15, 20, 30, 35, 40, and 45 days postbiotin injection. (a) Flow cytometric profiles showing expression of biotin and CD47 in RBCs at days 1, 5, 20, and 35 post‐biotinylation in WT (upper row) and *thbs1* ^−/−^ (lower row) were presented. Since newly produced RBCs from bone marrow are biotin^−^, so the changes in the biotin^+^ RBC population closely reflect the characteristics of aging RBCs. (b) Percentages of biotin^+^ RBCs in blood circulation of WT (*n* = 8) and *thbs1* ^−/−^ (*n* = 9) mice (mean ± *SEM*). (c, d) Direct STORM imaging of CD47 on biotin^−^ RBCs sorted from WT (c) or *thbs1* ^−/−^ (d) mice at day 30 post‐biotinylation (bar = 2 µm). Zoomed images (insets) highlight the extent of CD47 molecule aggregation on RBCs

To further determine the role of TSP‐1/CD47 binding in the clearance of aged RBCs, we compared the lifespan of RBCs between old WT and *thbs1* ^−/−^ C57BL/6 mice. The levels of biotin^+^ RBCs declined significantly slower in *thbs1* ^−/−^ than in WT mice, indicating that RBCs had a prolonged lifespan in mice lacking TSP‐1 (Figure 6a,b). Direct STORM imaging revealed that small aggregate CD47 clusters were seen in sorted biotin^−^ RBCs from WT, but not *thbs1* ^−/−^, mice at day 30 post‐biotinylation (Figure 6c,d), despite that there was no significant difference in the overall cluster characteristics of CD47 between these cells (Figure S3). These data indicate that TSP‐1 contributes to both the formation of CD47 aggregates on RBCs and the clearance of aging RBCs.

### 2.6 Role of lipid rafts in 4N1K‐induced CD47 rearrangement on RBCs from aged mouse

**Figure 7:**
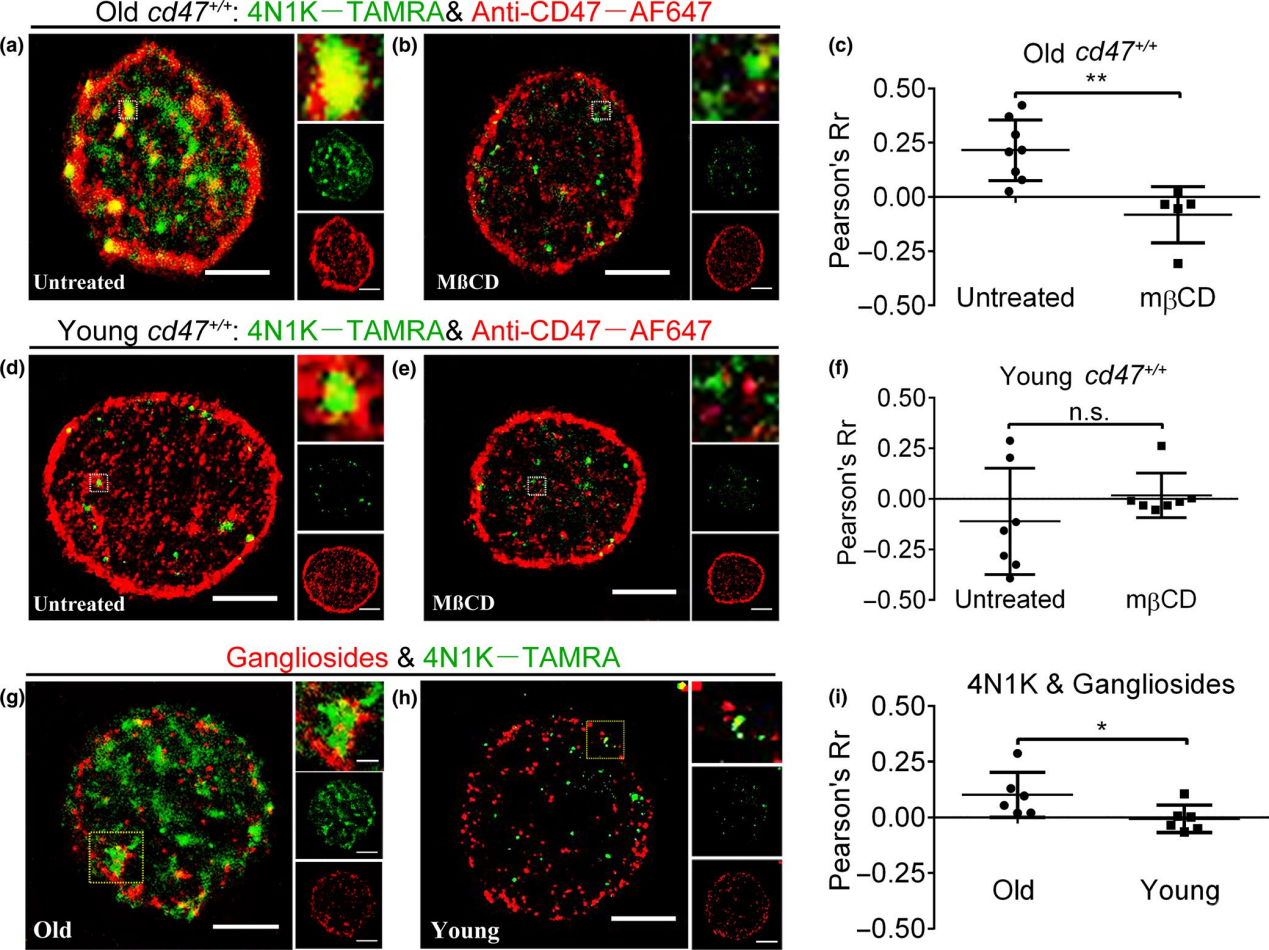
CD47 and 4N1K clustered in lipid rafts on young vs. old *cd47* ^+/+^ mouse RBCs. (a–f) RBCs from old (a–c) or young (d–f) *cd47* ^+/+^ mice were untreated or treated with MßCD, and analyzed by dual‐color dSTORM images using 4N1K–TAMRA and anti‐CD47–AF647. Shown are representative dSTORM images of untreated (a, d) and MßCD‐treated (b, e) RBCs (scale bar = 2 μm; zoomed images (insets) show co‐localized CD47 and 4N1K clusters; membrane‐bound 4N1K (green) and CD47 (red) distributions are also shown in the side row), and levels of co‐localization (Pearson's Rr) between 4N1K and CD47 on RBCs (c, f; *n* = 5–8 per group). (g–i) Analysis of 4N1K binding sites and gangliosides (a component of lipid raft) on RBCs. (g, h) Representative dual‐color dSTORM images showing 4N1K binding sites and CT‐B–AF647 labeled gangliosides on RBCs from old (g) or young (h) mice. Zoomed images (insets) show co‐localization between 4N1K and gangliosides. Membrane‐bound 4N1K (green) and gangliosides (red) distributions were also shown in the side row. (i) Levels of co‐localization between gangliosides and 4N1K on old (*n* = 6) or young (*n* = 8) mouse RBCs

Considering that CD47 exists in an anti‐detergent extraction component (McDonald, Zheleznyak, & Frazier, 2004) and co‐localizes with the lipid raft biomarker, stomatin (Rungaldier, Oberwagner, Salzer, Csaszar, & Prohaska, 2012), we examined whether lipid rafts are involved in 4N1K‐induced CD47 clustering. Briefly, RBCs were treated with methyl‐β‐cyclodextrin (MßCD) to disrupt lipid rafts (by extracting membrane cholesterol)(Fishman & Orlandi, 2003) and analyzed for 4N1K binding and CD47 distribution. 4N1K and CD47 were highly co‐localized in larger clusters on untreated, but not lipid raft‐disrupted old mouse RBCs (Figure 7a,b). Furthermore, RBCs appeared to be smaller after MßCD treatment (Figure S4). The degree of 4N1K/CD47 co‐localization on old mouse RBCs was reduced from a “moderate” level (Rr, 0.21 ± 0.11) to a “weak” level (Rr, 0.08 ± 0.13) after MßCD treatment (Figure 7c). In contrast, there was no significant binding or co‐localization between 4N1K and CD47 on young mouse RBCs, regardless of MßCD treatment (Figure 7d–f). These data suggest that lipid rafts participate in 4N1K‐induced CD47 clustering, which is required for binding to TSP‐1.

We further performed dual‐color dSTORM imaging to visualize the localization relationship between 4N1K and lipid rafts. We found that gangliosides (a component of lipid rafts) showed a random, dotted distribution pattern (red) on old mouse RBCs, while 4N1K showed the previously observed patched binding (green) (Figure 7g). The overlay images show clear co‐localization of 4N1K with ganglioside‐labeled lipid rafts (Figure 7g). However, no obvious co‐localization between 4N1K and gangliosides was detected in RBCs from young mice (Figure 7h). The level of co‐localization between 4N1K and gangliosides on old mouse RBCs (0.11 ± 0.09, “moderate”) was significantly greater than that on young mouse RBCs (−0.01 ± 0.06, “weak”) (Figure 7i). These data support the possibility that lipid rafts are involved in 4N1K‐induced CD47 clustering on old mouse RBCs.

## 3 DISCUSSION

Previous studies suggest that the role of CD47 in the clearance of aged RBCs might be attributable not only to downregulation of CD47, but also to its altered expression pattern and potential to bind TSP‐1(Burger et al., 2012). Here, we sought to identify aging‐associated changes in the spatial organization of CD47 proteins and in their ability to bind TSP‐1 using dSTORM technology that enables visualization of CD47 molecules at the nanoscale level. In young mouse RBCs, we found that CD47 molecules were evenly distributed as nanoclusters with very limited binding to TSP‐1. In contrast, old mouse RBCs showed a significant decrease in CD47 molecule numbers, but an increase in the density and average number of CD47 proteins within clusters. Despite the reduced number of CD47 molecules, the altered spatial organization of CD47 on old mouse RBCs was associated with markedly enhanced binding to TSP‐1. Furthermore, CD47 clusters on old mouse RBCs enlarged in size upon exposure to TSP‐1, while TSP‐1 exposure did not affect CD47 distribution on young mouse RBCs. Subsequent mechanistic studies revealed that lipid rafts are involved in TSP‐1‐induced CD47 clustering on old mouse RBCs. These findings provide direct evidence that CD47 molecules on RBCs show decrease in number and change in conformation during the aging process, with the latter leading to enhanced binding to TSP‐1 as well as TSP‐1/CD47 interplay.

As a marker of self, CD47 inhibits phagocytosis by signaling through the inhibitory receptor SIRPα. CD47 is also a receptor for TSP‐1, and its signaling plays a complex role in regulation of cell function and survival (Brown & Frazier, 2001; Isenberg et al., 2009; Soto‐Pantoja, Ridnour, Wink, & Roberts, 2013). Our results unexpectedly revealed that there is only minimal or limited binding of TSP‐1 to CD47 on young RBCs. We further found that the binding strength of TSP‐1 to CD47 is predominantly determined by the distribution pattern and not by the amount of CD47 on RBCs. These findings suggest that TSP‐1/CD47 signaling may play a minimal role in normal cells, but have a significant impact on diseased cells, such as senescent and apoptotic cells.

Previous studies using CD47‐deficient mice raised the possibility that CD47 downregulation on aged RBCs is an important mechanism for their clearance by macrophages (Oldenborg et al., 2000), but direct evidence has been lacking. Using dSTORM imaging and qSMLM analysis, we found that the numbers of CD47 molecules on old mouse RBCs were similar to those of young *cd47* ^+/−^ RBCs. Because the level of CD47 expression on *cd47* ^+/−^ mouse cells (approximately half the level expressed by *cd47* ^+/+^ cells) is sufficient for protection against phagocytosis, even in an allogeneic setting (Wang, Wu, Wang, Oldenborg, & Yang, 2010), factors other than CD47 downregulation are likely involved in mediating phagocytosis of old RBCs. In support of this possibility, TSP‐1 binding to CD47 on aged RBCs was reported to promote phagocytosis (Burger et al., 2012). In this study, we found that CD47 clustering on old RBCs is an important factor that strengthens CD47 binding to TSP‐1 and that TSP‐1/CD47 engagement can further induce CD47 molecule reorganization into larger clusters. Furthermore, aged RBCs showed significantly prolonged survival or slowed clearance in *thbs1* ^−/−^ mice, providing further evidence supporting the role of TSP‐1/CD47 binding in the removal of aging RBCs. Taken together, these studies indicate that a change in CD47 distribution on aged RBCs is a major factor enhancing CD47 binding to TSP‐1, hence facilitating phagocytosis. TSP‐1 was also reported to mediate the clearance of apoptotic senescent neutrophils via interaction with CD36 and α_v_β_3_ integrin (Savill, Hogg, Ren, & Haslett, 1992). Given that CD47 is a high‐affinity receptor for TSP‐1 and closely associated α_v_β_3_ expression and function (Soto‐Pantoja, Kaur, & Roberts, 2015), CD47 may also be involved in TSP‐1‐induced phagocytosis of apoptotic neutrophils. Although our findings on RBCs may not directly apply to other types of cells due to their different macromolecular complexes, the super‐resolution imaging approach used in this study offers an effective means of understanding how the molecular context of CD47 on other cell types modulates its interactions with TSP‐1.

Although the role of lipid rafts in CD47 signaling remains to be determined, CD47 and lipid raft proteins are present in RBC‐derived vesicles (Kriebardis et al., 2008; Rungaldier et al., 2012). In the present study, we show that 4N1K binding sites on old mouse RBCs were co‐localized with gangliosides (a component of lipid rafts) and that TSP‐1/4N1K failed to bind CD47 on old mouse RBCs after treatment with MßCD. These results indicate that the mobility of lipid rafts is involved in TSP‐1‐induced CD47 redistribution on old RBCs. In support of our findings, it has been reported that TSP‐1 regulates cell spreading or platelet activation via CD47 in a pertussis toxin‐sensitive way (Chung, Gao, & Frazier, 1997; Gao et al., 1996).

In summary, this study provides clear nanoscale pictures of CD47 distribution, TSP‐1/CD47 binding, and associated changes on RBCs from young and aged mice. On healthy RBCs, CD47 molecules reside as nanoclusters with minimal binding to TSP‐1. On aged RBCs, CD47 proteins form bigger and denser clusters and gain an increased ability to bind TSP‐1, despite a decrease in total CD47 molecules. Moreover, TSP‐1/CD47 binding results in a further increase in the size of CD47 clusters in a process that involves lipid raft aggregation. These observations provide new insights into aging‐associated changes in CD47 on RBCs, and a molecular basis for understanding how CD47, TSP‐1, and SIRPα interact to remove aged RBCs.

## 4 EXPERIMENTAL PROCEDURES

### 4.1 Animals and key reagents

The *cd47* ^+/+^ wild‐type, *cd47* ^−/−^ C57BL/6 (B6) and *thbs1* ^−/−^ (B6) mice were purchased from the Jackson Laboratory; CD47 heterozygous (*cd47* ^+/−^) B6 mice were from the F1 generation of *cd47* ^+/+^ and *cd47* ^−/−^ B6 mice. Mice were housed in a specific pathogen‐free microisolator environment. All animal studies were approved by the Institutional Animal Care and Use Committee of the First Hospital of Jilin University, and all experiments were performed in accordance with protocol. Alexa Fluor 647 (AF647)‐conjugated rat anti‐mouse CD47 (clone Miap301) was purchased from BioLegend. TSP‐1 peptide (4N1K, amino acid sequence: KRFYVVMWKK) and control peptide (4NGG, amino acid sequence: KRFYGGMWKK) conjugated with TAMRA were synthesized by Sangon Biotech (Shanghai). Recombinant mouse TSP‐1 was purchased from R&D systems. Recombinant cholera toxin subunit B conjugated with Alexa Fluor 647 was ordered from Invitrogen.

### 4.2 Functionalized cover glass

APTES functionalized cover glass was prepared as previously described (Pan et al., 2014). Briefly, a desiccator containing dry and cleaned cover glass (Fisherbrand) was purged with argon for 2 min, and 50 μl of APTES (aminopropyltriethoxysilane, 99%, Sigma‐Aldrich, St. Louis, MO) was placed into a small container at the bottom of the desiccator. A 15 μl volume of N,N‐diisopropylethylamine (99%, distilled, Sigma‐Aldrich) was placed into another small container, and the desiccator was purged with argon for an additional 5 min and then sealed off, leaving the cover glass exposed to APTES vapor overnight. The treated cover glass (APTES‐cover glass) was stored in the sealed desiccator until use.

### 4.3 Preparation of RBCs

Mouse RBCs were collected from peripheral blood, washed three times with saline‐adenine‐glucose‐mannitol (SAGM, 150 mM sodium chloride, 1.25 mM adenine, 50 mM glucose, 29 mM mannitol), and re‐suspended in SAGM (Burger et al., 2012). Cell concentrations were determined by cell counting.

### 4.4 Staining of RBCs for dSTORM imaging

To investigate the distribution of CD47 at the molecular level, RBCs were stained with AF647‐labeled anti‐CD47 mAb (labeling ratio was between 0.5–1, determined by UV‐spectrophotometer) attached to the APTES‐modified cover glass, and visualized with dSTORM(Rust, Bates, & Zhuang, 2006).

For dSTORM imaging of CD47, RBCs were incubated with blocking buffer containing 3% BSA for 30 min at room temperature, then stained with a saturated concentration of anti‐CD47–AF647 at room temperature for 1 h, and washed three times with PBS. For dual‐color imaging of 4N1K or TSP‐1 binding and CD47 distribution, RBCs were blocked with isotype control antibodies, then left untreated or treated with 1 mM methyl‐β‐cyclodextrin (MβCD) for 30 min to extract cholesterol. The RBCs were first labeled with 4N1K–TAMRA (100 nM), 4NGG–TAMRA (100 nM), or TSP‐1–TAMRA (2.2 nM)(Bauer et al., 2012) for 30 min, washed three times with PBS, and then stained with anti‐CD47–AF647 for 1 h. For dual‐color imaging of 4N1K binding and lipid rafts, recombinant cholera toxin subunit B conjugated with Alexa Fluor 647 was used to identify gangliosides in the lipid rafts(Fishman & Orlandi, 2003). The RBCs were blocked with BSA, labeled with 4N1K, and then stained with recombinant cholera toxin subunit B‐AF647 (at 1 μg/mL) for 1 h prior to imaging. After staining, RBCs were plated onto functionalized cover glass for 20 min and incubated with 4% paraformaldehyde supplemented with 0.2% glutaraldehyde in PBS for 10 min. Diluted (100 nm) tetra‐spectra micro‐beads (Life Technologies) were placed on each sample for two‐channel alignment and drift correction. Before sealing the cover glass with nail polish, imaging buffer was added as previously described (Yan et al., 2018).

### 4.5 Direct STORM imaging

Direct STORM images were acquired on a total internal reflection fluorescence microscope (Nikon, Tokyo, Japan) with a 100× oil‐immersion objective with a numerical aperture of 1.49. For AF647, 100 mW of 633 nm laser illumination was used for imaging. For TAMRA, 40 mW of 532 nm laser illumination was used. For dSTORM imaging, 10,000–20,000 frames were acquired per sample with a cooled, electron‐multiplying charge‐coupled device camera (Photometric CascadeII) with an exposure time of 30 msec. For two‐color images, the Alexa Fluor 647 channel was acquired first, followed by TAMRA. During the acquisition period, the z‐drift was eliminated by a focus lock. Recorded images were preliminarily reconstructed with the quick PALM Image J plug‐in (Henriques et al., 2010), and quantitatively analyzed with another ImageJ plug‐in, GDSC SMLM software(Herbert, 2014).

### 4.6 Data analysis

To extract quantitative parameters, raw data from dSTORM imaging were reconstructed with the GDSC SMLM (Herbert, 2014) ImageJ plug‐in. Briefly, peak fitting parameters (calibration, gain, exposure time) were input as experimental settings. The fluorescence peaks were identified in each frame, and least‐squared‐error was selected as fit criteria in the Gaussian fitting function. The signal strength was set as 60 and precision at a 50 nm cutoff in peak filtering. Fourier image resolution was calculated with updated results after drift calculation. Quantitative data were extracted using GDSC SMLM (Gao, Chen, Gao, Wang, & Xiong, 2017; Paparelli, Corthout, Pavie, Annaert, & Munck, 2016; Salehi‐Reyhani, 2017). “Dark time analysis” and “blink estimator” were performed for the fluorescent probes, and substituted into subsequent analysis such as “trace molecules” and “cluster molecules.” The dark time and blink rate of the fluorescent probe was calculated to exclude re‐excited fluorescent probes (Annibale et al., 2011). The expected number of molecules is equal to the observed number of pulses divided by the average blinking rate of a fluorophore (Coltharp, Kessler, & Xiao, 2012), which can be extracted directly from the log file.

To analyze the spatial organization of CD47 and 4N1K during aging, a function in the PC‐PALM subset was used. In the PC‐PALM fitting plug‐in, the pair correlation function was used to quantify spatial organization (Sengupta et al., 2011, 2013; Veatch et al., 2012). Three parameters were extracted from PCF analysis: domain radius (radius of the clusters), N^cluster^ (average number of molecules in a cluster), ψ^cluster^ (local domain density increment in a cluster, calculated as the ratio of the cluster protein density to the overall protein density of the membrane area) (Notelaers et al., 2014). The degree of co‐localization was calculated with the ImageJ plug‐in, “Co‐localization finder.” According to the values of Pearson's correlation coefficient (Rr) (Zinchuk, Wu, & Grossenbacher‐Zinchuk, 2013), the co‐localization degree can be classified as “very weak” (Rr, −1.0~−0.27), “weak” (Rr, −0.26 ~ 0.09), “moderate” (Rr, 0.1–0.48), “strong” (Rr, 0.49–0.84), or “very strong” (Rr, 0.85−1.0). Relative quantitative data extracted using GDSC SMLM or “Co‐localization finder” in an unbiased manner were further analyzed using GraphPad Prism version 6.02 for Windows software.

### 4.7 Analysis of RBC aging and clearance in vivo by biotinylation assay

We used in vivo biotinylation method as previously described (Khandelwal et al., 2007; Saxena et al., 2012). Briefly, 2‐month‐old or 18‐month‐old *cd47* ^+/+^ mice and *thbs1* ^−/−^ mice were given three daily injections of Biotin‐X‐NHS ester (270 µl per injection, freshly prepared by mixing 20 µl stock solution and 250 µl PBS; stock solution, was prepared by dissolving 25 mg Biotin‐X‐NHS ester in 500 µl of dimethylformamide). After biotinylation, blood cells were collected at different time points and analyzed for percentages of biotin^+^ RBCs by staining with V500‐conjugated streptavidin using flow cytometry, and the rate of decline in biotin^+^ cells reflects the life span of labeled RBCs.

### 4.8 Flow cytometry analysis

Blood was collected from biotin‐injected mice, diluted (1:100) in PBS (5 mM EDTA), and the diluted sample (equivalent to 0.1 µl of blood) was stained with V500‐conjugated streptavidin and Alexa Flour 647‐conjugated anti‐CD47 mAb (clone Miap301). When analyzing a mixture of samples from two different (e.g., young and old) mice, one sample stained with anti‐Ter119‐FITC before mixing the samples and staining with V500‐streptavidin and Alexa Flour 647‐anti‐CD47 mAb. After incubation for 30 min at 4°C, cells were washed and analyzed on a flow cytometer (Fortessa, BD biosciences). Data were analyzed with FlowJo_V10.

### 4.9 Statistical analysis

Statistical analysis was performed using GraphPad Prism 6.0. Differences between group means were analyzed by unpaired two‐tailed Student's *t*‐test or repeated‐measure (RM) two‐way ANOVA. Where there were more than two groups, differences among group means were analyzed by one‐way ANOVA test with post hoc analysis by Tukey's test. A *P* value of <0.05 was considered significant. Figures produced by ImageJ or GraphPad were arranged with Adobe Photoshop CS3.

## CONFLICT OF INTEREST

The authors declare no competing financial interests.

## AUTHOR CONTRIBUTIONS

F.W., Y‐H.L., H.W., and Y‐G.Y. designed experiments; F.W., Y‐H.L., J.G., T.Z. G‐Y.X. performed experiments; F.W., J.G., H.W., and Y‐G.Y. analyzed data; Y.X. performed dSTORM imaging; W‐J. Z. performed FACS sorting; F.W., H.W. and Y‐G.Y. wrote the paper; all authors edited and approved the manuscript.

## Supporting information

 * Click here for additional data file.

## Data Availability

The data that support the findings of this study are available from the corresponding authors upon reasonable request.
