# Supplementary material for: Aging‐associated changes in CD47 arrangement and interaction with thrombospondin‐1 on red blood cells visualized by super‐resolution imaging
Source: Aging Cell. 2020 Aug 31;19(10):e13224. doi: 10.1111/acel.13224 (PMC7576236; doi:10.1111/acel.13224)
Supplement: Supplementary file 1 — * [file ACEL-19-e13224-s001.pdf]

Supplementary Figures

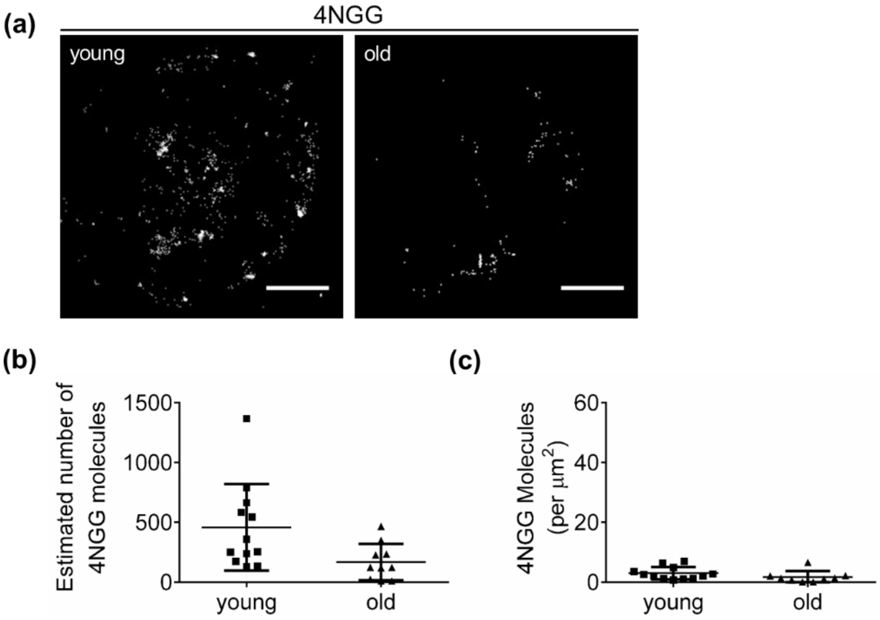

Figure S1. Direct STORM imaging and quantitative analysis of 4NGG binding on young and old *cd47*$^{+/+}$ mouse RBCs. (**a**) Representative dSTORM images of 4NGG binding on young and old *cd47*$^{+/+}$ mice RBCs. Scale bar = 2 µm. (**b-c**) Quantitative analysis of 4NGG binding on young and old *cd47*$^{+/+}$ mice RBCs. Data shown are estimated numbers (**b**), protein densities (molecules/µm^2) (**c**) of 4NGG molecules on RBCs.

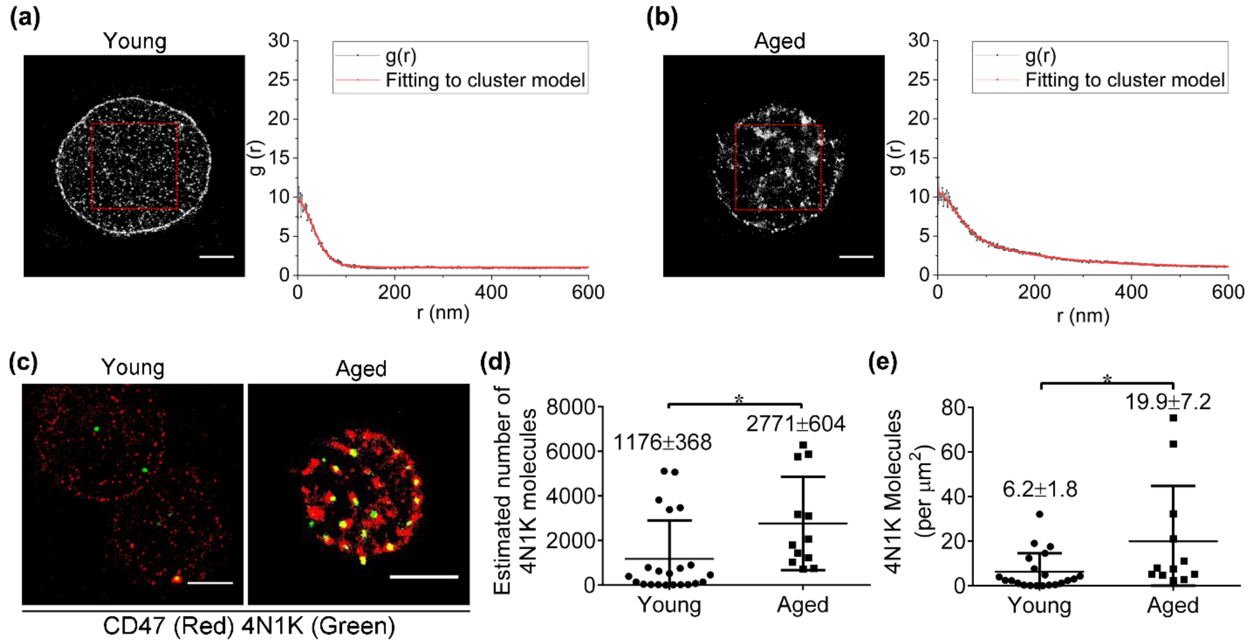

Figure S2. CD47 clustering and 4N1K binding on young vs. aged RBCs from young *cd47*$^{+/+}$ mice. Young RBCs (biotin^{-}) and aged RBCs (biotin^{+}) were sorted from young (2-month-old) mice 20 days after injection of Biotin-X-NHS ester and examined by dSTORM imaging. (**a, b**) Left and right panels show RBC dSTORM images (scale bar = 2 μm) and corresponding pair correlation function analysis, respectively, for sorted young (biotin^{-}) (**a**) and aged (biotin^{+}) (**b**) RBCs. (**c-e**) Two-color dSTORM imaging and quantitative analysis of 4N1K binding on young and aged RBCs. (**c**) Representative dSTORM images showing co-localization of 4N1K (green) with CD47 (red) on young and aged RBCs. Scale bar = 2 μm. (**d, e**) Estimated numbers (**d**) and protein densities (molecules/μm^2) (**e**) of 4N1K molecules on young and aged RBCs. Data are combined from two independent experiments and presented as mean ± SD; *, $P < 0.05$ (unpaired *t*-test).

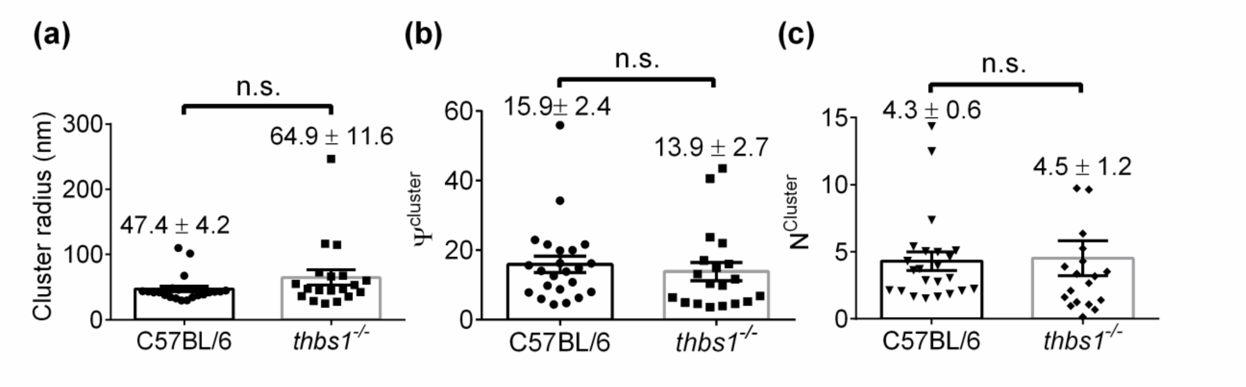

Figure S3. Clustering parameters of CD47 clusters on sorted biotin⁻ RBCs cohorts from 18-months of age C57BL/6 and *thbs1*$^{-/-}$ mice. (**a**) CD47 cluster radius (nm); (**b**) density of CD47 proteins in cluster ($\psi^{cluster}$); (**c**) average numbers of CD47 proteins per cluster ($N^{cluster}$). Unpaired *t*-test was used, n.s. indicates not significant; Data are combined from 2 independent experiments and presented as mean ± SD (each symbol represents an individual cell; ~20 cells per group were reanalyzed).

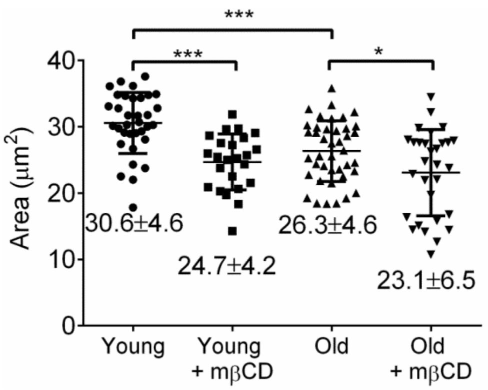

Figure S4. MßCD treatment reduces the size of mouse RBCs. Mouse (*cd47*$^{+/++}$) RBCs from young and old mice were untreated or treated with MßCD, then plated on the APTES-cover glass, and areas of individual RBCs were measured by ImageJ. Data shown are the single side areas (mean ± SDs; per cell) of untreated young (n=34) and old (n=41) RBCs, and MßCD-treated young (n=24) or old (n=30) RBCs. Each symbol represents an individual cell. * $P < 0.05$, ** $P < 0.01$ and *** $P < 0.0005$ (*t*-test)
